# *Nymphaea* “Eldorado” flower extract targets serpine 1 to attenuate inflammatory and antioxidant crosstalk in zebrafish

**DOI:** 10.3389/fphar.2025.1612233

**Published:** 2025-07-11

**Authors:** Liyan Mao, Lingyun Long, Qian Qin, Qiuwei Huang, Qiulan Huang, Yanping Yu, Liqiong Ding, Gongde Liu, Ji Zhang, Beimi Cui, Xiaohui Tan

**Affiliations:** ^1^ Guangxi Subtropical Crops Research Institute, Guangxi Academy of Agricultural Sciences, Nanning, China; ^2^ Guangxi Key Laboratory of Quality and Safety Control for Subtropical Fruits, Guangxi Subtropical Crops Research Institute, Nanning, China; ^3^ Institute of Molecular Plant Sciences, School of Biological Sciences, University of Edinburgh, Edinburgh, United Kingdom

**Keywords:** *Nymphaea* “Eldorado”, anti-inflammatory, antioxidant, zebrafish, transcriptomics, molecular docking

## Abstract

Chronic inflammation and oxidative stress are pivotal drivers of pathological conditions, necessitating safer plant-derived therapeutic alternatives. This study elucidates the anti-inflammatory and antioxidant mechanisms of *Nymphaea* “Eldorado” flower water extract (NEWE) in a zebrafish (*Danio rerio*) model of copper sulfate (CuSO_4_)-induced inflammation. NEWE (25–100 μg/mL) attenuates CuSO_4_-triggered neutrophil migration and oxidative stress by downregulating proinflammatory genes (*il1b*, *ptgs2a/b*) and reducing reactive oxygen species (ROS) production. Transcriptomic profiling identified 339 differentially expressed genes (DEGs), enriched in cytokine signaling and redox regulation, with *serpine1*, *stat3*, and *mmp9* emerging as key regulatory hubs. Widely targeted metabolomics revealed 891 bioactive compounds, including flavonoids and phenylpropanoids, with network pharmacology predicting multi-target interactions involving inflammatory and oxidative stress pathways. Molecular docking confirmed binding affinities between protocatechuic acid, L-pyroglutamic acid, and Serpine1’s active site, indicating direct interference with inflammation modulation. Collectively, these results establish NEWE as a polypharmacological agent that disrupts inflammation-oxidative stress crosstalk primarily through Serpine1-mediated pathways, offering a molecular foundation for plant-derived interventions against chronic inflammatory diseases.

## 1 Introduction

Inflammation and oxidative stress are hallmarks of chronic diseases. Chronic inflammation can lead to tissue damage and pathological conditions such as cardiovascular disease, diabetes, and cancer (Dhabhar, 2014; Edlow et al., 2022; Garbarino et al., 2021; Porter et al., 2020; Prata et al., 2024). Oxidative stress is caused by the disruption of redox homeostasis, causing excessive reactive oxygen species (ROS) production, cellular damage, and systemic inflammation (Jin and Kang, 2024; Vo et al., 2011), potentially leading to chronic diseases, including neurodegenerative disorders and metabolic syndrome (Lee et al., 2024). Environmental factors, poor diet, and chronic stress exacerbate these processes, heightening the risk of disease development (Lee et al., 2024; Prata et al., 2024). Nonsteroidal anti-inflammatory drugs and corticosteroids are commonly used to reduce inflammation (Ju et al., 2022; Sapieha et al., 2011; Zhou et al., 2014). However, the long-term use of these medications may cause adverse effects, including gastrointestinal bleeding, acute kidney injury, and increased cardiovascular risk (Atiquzzaman et al., 2019; Laine, 2001; Misurac et al., 2013). These detrimental effects underscore the urgent need to develop alternative treatments with plant-based anti-inflammatory and antioxidant compounds, aiming to minimize toxicity and the incidence of adverse effects (Haj et al., 2015; Leu et al., 2019).

Water lilies (Nymphaea L. Nymphaeaceae) are a rich source of bioactive compounds, including flavonoids, alkaloids, and phenols, exhibiting diverse therapeutic effects, including antimicrobial and antioxidant activities (Hsu et al., 2013; Ren et al., 2019; Sheichenko et al., 2019). Thus, determining the pharmacological activities and mechanisms of action of Nymphaea extracts remains to be investigated to explore their potential as anti-inflammation and anti-oxidative stress agents.

Zebrafish (*Danio rerio*) is a valuable model organism for natural product research to its genetic and physiological conservation with humans, rapid development, and transparent embryos that allow the visualization of biological processes and the trafficking of fluorescent cells *in vivo* (Howe et al., 2013; Postlethwait et al., 1998). Zebrafish have unique advantages over rodent models, including higher fecundity, cost-effectiveness, and suitability for high-throughput screening (Ali et al., 2011; Choi et al., 2021). Thus, zebrafish models are ideal for evaluating the pharmacological properties of plant compounds. Specifically, these models are used to assess the ability of plant compounds to modulate the copper sulfate (CuSO_4_)-induced activation and recruitment of fluorescently labeled neutrophils, thereby accelerating the discovery and development of new therapeutics (Singh et al., 2022; Xie et al., 2020; Zhang et al., 2023a).

This study investigated the anti-inflammatory and antioxidant effects of water lily (*Nymphaea* “Eldorado”) flower water extract (NEWE) using a zebrafish model of copper sulfate (CuSO_4_)-induced inflammation. Genes and metabolic pathways regulated by extract components were identified by transcriptomic and metabolomic analyses. Potential molecular targets of bioactive compounds were identified by network pharmacology and molecular docking. The results offer valuable insights into the mechanisms of action of NEWE, supporting its potential therapeutic use in reducing inflammation and oxidative stress to improve human health and wellbeing.

## 2 Materials and methods

### 2.1 Sample extraction

Fresh flowers of *Nymphaea* “Eldorado” were sourced from the Liuzhou Shixian Planting and Breeding Cooperative (Liuzhou, China). Fresh flowers on the first day of flowering were collected and washed with water (Supplementary Figure S1A). The flowers were placed in a container filled with water and exposed to daylight for 24 h. The flowers were opened, and stalks and sepals were removed. The ovary was cut by 1/3, and the flowers were dried at 70°C–80°C until the moisture content was less than 10% (Supplementary Figure S1B). The dried flowers were crushed in a grinder, and the powder was sieved through a 20-mesh sieve and stored at −20°C for later use.

The extract (5,120 mg) was placed in a 1 L flask and mixed with 400 mL of water (yielding a solid-to-solvent ratio of 1:78, w/v) at 100°C. After standing for 30 min at room temperature, the mixture was centrifuged. The supernatant was collected, and the pellet was resuspended in 400 mL of water at 100 C. After standing for 30 min, the sample was centrifuged as described above. The supernatant was collected and combined with the first supernatant. The stock solution (6,400 mg/L) was stored at −20°C.

### 2.2 Toxicity assessment

The stock solution was diluted to obtain working concentrations (100, 200, 400, 800, 1600, 3200, and 6,400 mg/L) (Supplementary Figure S1). At 72 h post-fertilization (hpf), AB strain wild-type zebrafish embryos were placed in 24-well plates with 10 embryos per well and three replicates per concentration (Supplementary Figure S1C). The culture medium was removed, and 2 mL of NEWE was added to each well. Based on the results of these assays, NEWE concentrations that were not toxic to zebrafish embryos were used in subsequent experiments (Supplementary Figure S1). All procedures involving animals were approved by the Animal Care and Use Committee of the Guangxi Academy of Agricultural Sciences and complied with Chinese government regulations.

### 2.3 Analysis of neutrophil migration

This study used the transgenic zebrafish line *Tg(mpx:GFP)*, which expresses GFP under the neutrophil-specific myeloperoxidase promoter, as described previously (Renshaw et al., 2006). Zebrafish embryos were placed in 90 mm dishes containing 1×E3 medium (pH was adjusted to 7.0–7.2 using 5 mM NaCl, 0.33 mM CaCl_2_, 0.17 mM KCl, and 0.33 mM MgSO_4_) with 100 embryos per dish. At 24 hpf, dead embryos were removed, and healthy embryos were added. At 72 hpf, normal embryos were transferred to 24-well culture plates with 10 embryos per well and incubated with 20 μM CuSO_4_ + NEWE (0, 25, 50, 75, 100 μg/mL) for 2 h at 28°C ± 1°C to induce neutrophil migration. GFP-expressing neutrophils in the anterior yolk sac were observed under a fluorescence microscope (Axio Imager M2, Zeiss) at 2 h after CuSO_4_ + NEWE treament to evaluate the degree of inflammation.

### 2.4 Measurement of antioxidant activity

WT zebrafish embryos were incubated with NEWE (0, 25, 50, 75, 100, 1255 μg/mL) or 100 μM quercetin (positive control) for 1 h at 28°C ± 1°C, followed by treatment with 10 μM CuSO_4_ for 20 min as described in Section 2.3. After washing with culture medium, embryos were incubated with 20 μg/mL of the fluorescent indicator 2′,7′-dichlorodihydrofluorescin diacetate (DCFH-DA) for 1 h at 28°C ± 1°C. CuSO_4_-induced ROS in embryos was detected by the formation of the oxidized compound dichlorofluorescein by fluorescence microscopy (Axio Imager M2, Zeiss) (Andoh et al., 2006; Girard-Lalancette et al., 2009; Zhang et al., 2023a; Zhang et al., 2023b).

### 2.5 RNA extraction and quantitative real-time polymerase chain reaction (qRT-PCR)

*Tg(mpx:GFP)* zebrafish embryos were incubated with 20 μM 20 μM CuSO_4_+NEWE 2 h at 28°C ± 1°C as described in Section 2.3. RNA from each sample (50 μg/mL) was extracted using TRIzol and reverse transcribed using the PrimeScript RT reagent kit. qRT-PCR was performed using the TB Green Premix Ex Taq II reagent kit (TaKaRa, Dalian, China) on the LightCycler 480 II System (Roche, Indianapolis, IN, United States). The cycling conditions were as follows: initial denaturation at 95°C for 30 s, followed by 40 cycles of 95°C for 5 s and 60°C for 20 s. Melting curves were generated by holding at 95°C for 5 s and 65°C for 1 min and increasing to 95°C at a rate of 0.1°C per second. The primers used in this study are listed in Supplementary Table S1. Relative gene expression levels were quantified using the 2^−ΔΔCT^ method (Livak and Schmittgen, 2001). The data were visualized using Origin 2021 (OriginLab, Northampton, MA, United States).

### 2.6 Library construction and RNA-seq analysis

*Tg(mpx:GFP)* zebrafish embryos were cultured as described in Section 2.3. Three treatment groups were established: control (untreated), model (treated with CuSO_4_), and treatment (treated with 20 µM CuSO_4_ + 50 μg/mL NEWE).

The construction of sequencing libraries and RNA-seq were performed using the Illumina HiSeq platform (Biomarker Biotechnology Corporation Co., Beijing, China). High-quality reads were assembled using StringTie. Genes with log2 fold-change >2.0 or < −2.0 and false discovery rate (FDR) < 0.01 were considered differentially expressed genes (DEGs). DEGs were identified using edgeR (https://bioconductor.org/packages/release/bioc/html/edgeR.html). Relative transcript abundance was measured as fragments per kilobase of exon per million fragments mapped using Cuffdiff, with an FPKM threshold set at a minimum of 1.0 to ensure the reliability of expression levels. Histograms, scatter plots, volcano plots, Venn diagrams, and heatmaps were generated on the Biomarker Cloud Platform (Biomarker Biotechnology Corporation Co., Beijing, China) or https://hiplot.com.cn/cloud-tool/drawing-tool/list.

### 2.7 Widely targeted metabolomic analysis

The stock solution was diluted to 50 mg/L in culture medium. Metabolomics analyses were performed by MetWare Biotechnology (Wuhan, China). Metabolite extraction, identification, and quantification were performed as described previously (Yuan et al., 2018). Samples were freeze-dried, ground into powder, and dissolved in 70% methanol as internal standard (1:30 v/v). The samples were centrifuged at 12,000 rpm for 3 min at 4 C, and the supernatant was filtered through a 0.22 μm membrane for analysis.

Metabolites were analyzed by ultra-performance liquid chromatography (Exion LC AD System, AB Sciex, Darmstadt, Germany) coupled with electrospray tandem mass spectrometry (ESI-Q TRAP-MS/MS (AB SCIEX Q TRAP 4000, Applied Biosystems, Foster City, CA, United States). Chromatographic separation was performed on an SB-C18 column (1.8 µm, 2.1 mm × 100 mm, Agilent). Elution was carried out using a system consisting of solvent A (0.1% formic acid in water) and solvent B (0.1% formic acid in acetonitrile) and a linear gradient from 95% A to 95% B in 9 min, 95% B for 1 min, 95% B to 95% A in 1.1 min, and 95% A in 2.9 min. The flow rate was 0.35 mL/min at 40°C, and the injection volume was 2 μL. The ESI conditions were as follows: source temperature, 550 C; ion spray voltage (IS), 5500 V (positive mode)/-4500 V (negative mode); gas pressure of 50, 60, and 25 psi for GS1, GS2, and CUR gases, respectively. The collision-activated dissociation was set to high. Multiple reaction monitoring mode was utilized for metabolite detection, with declustering potential and collision energy individually optimized for each transition.

Metabolites abundance was categorized into two groups (classes I and II) based on peak areas.

### 2.8 Prediction of target genes and network construction

Genes potentially targeted by metabolites were identified using the Traditional Chinese Medicine Systems Pharmacology Database and Analysis Platform (https://old.tcmsp-e.com/tcmsp.php) and SwissTargetsPrediction (http://www.swisstargetprediction.ch/). Drug-target networks integrating transcriptomic and metabolomics data were constructed using Cytoscape version 3.9.1 (Li et al., 2022).

### 2.9 Molecular docking

The molecular structure of L-pyroglutamic acid, monomyristin, and protocatechuic acid were obtained from the PubChem database (https://pubchem.ncbi.nlm.nih.gov/). The 3D structure of the target protein, Serpine1, also known as plasminogen activator inhibitor-1, was obtained from the Protein Data Bank (https://www.rcsb.org/structure/4DTE). Protein-metabolite interactions were predicted using AutoDock Tools version 1.5.7, which applies a semi-flexible docking approach to predict binding affinities by exploring ligand and receptor flexibility. The docking grid was centered on the protein’s active site, with grid spacing and box size parameters optimized to encapsulate potential binding pockets. For each ligand, binding modes were generated based on the lowest binding energy utilizing the Lamarckian Genetic Algorithm as the primary search strategy, which has been validated in several studies for its efficacy in docking simulations. The AutoDock results were visualized and analyzed using PyMOL version 4.6.0 (Morris et al., 2009; Seeliger and de Groot, 2010), allowing detailed observation of ligand binding conformations, hydrogen bonding, and hydrophobic interactions, providing insights into the stability and specificity of ligand binding to Serpine1.

### 2.10 Statistical analysis

Data normality was assessed using the Kolmogorov–Smirnov test. Normally distributed continuous variables were expressed as mean ± standard deviations, and the significance of differences between means was determined using paired Student’s t-test or one-way analysis of variance followed by Tukey’s *post hoc* test. Differences between groups were compared using the Kruskal–Wallis test and Dunn–Sidak multiple comparison test. Box plots were drawn using Origin 2021 (OriginLab, Northampton, MA, United States). *P*-values of less than 0.05 were considered statistically significant.

## 3 Results

### 3.1 Effect of NEWE on CuSO_4_-induced inflammation in zebrafish larvae

**FIGURE 1:**
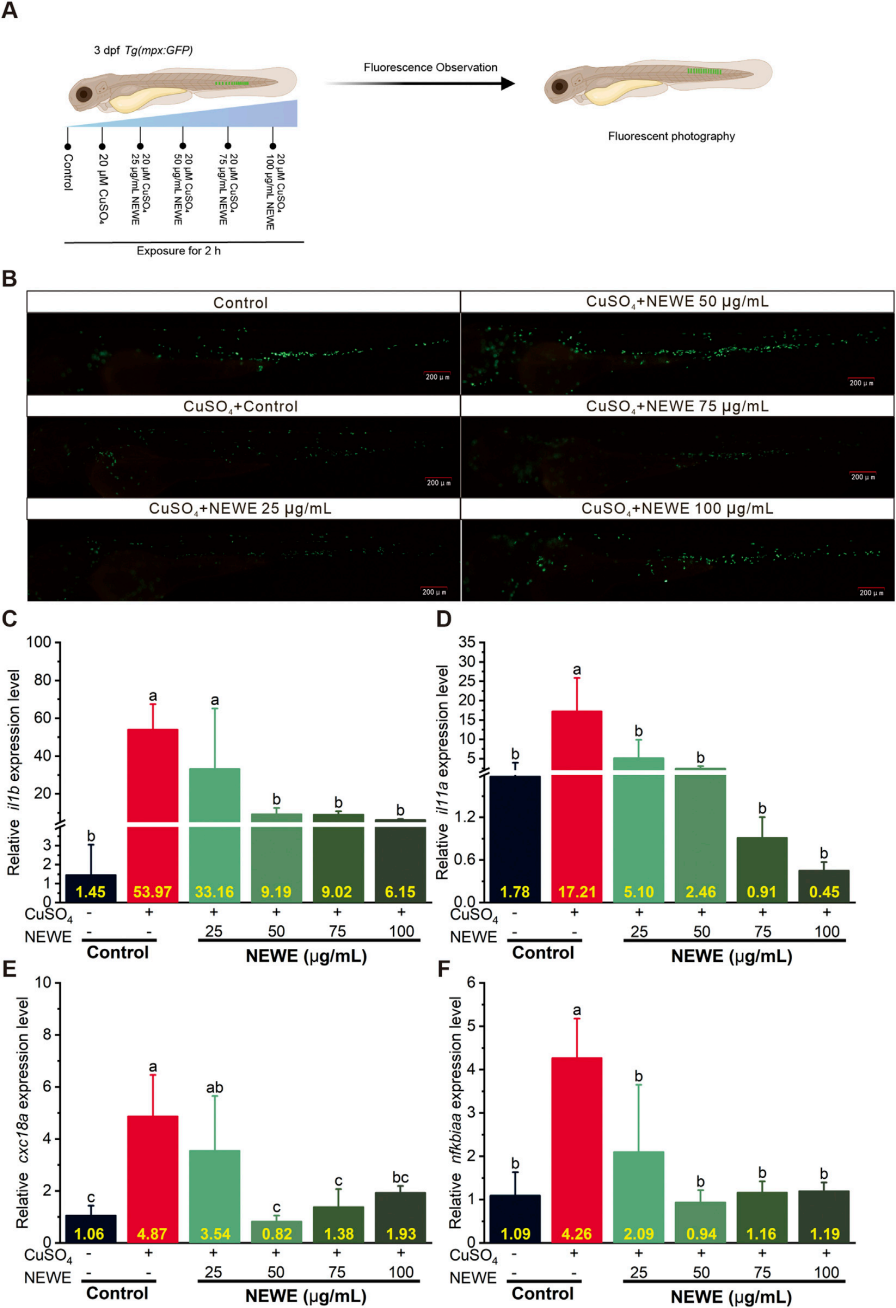
Effects of *Nymphaea* “Eldorado” flower water extract (NEWE) on CuSO_4_-induced inflammation in zebrafish larvae. **(A)** Flowchart of the experiments. **(B)** Fluorescence analysis of neutrophil migration in zebrafish larvae treated with CuSO_4_ in the presence or absence of NEWE. In the control group, neutrophils were localized predominantly to the caudal hematopoietic tissue in the ventral trunk and tail region. Upon CuSO_4_ treatment, neutrophils migrated towards the horizontal midline and accumulated near lateral line neuromasts, indicating an inflammatory response. Treatment with various concentrations of NEWE significantly inhibited CuSO_4_-induced neutrophil migration to inflammatory sites, suggesting an anti-inflammatory effect. GFP-expressing neutrophils are shown in green. Scale bar: 200 µm. **(C–F)** qRT-PCR analysis of the relative expression of inflammatory genes in zebrafish larvae. Gene expression was normalized to β-actin. Data are means ± SDs of three independent experiments. Different letters indicate significant differences (*p* < 0.05, Student’s t-test).

Acute toxicity assessment revealed that the 24-h LC_50_ of NEWE for 72 h post-fertilization (hpf) zebrafish embryos was 3200 μg/mL (Supplementary Figure S1D). Based on this safety profile, we evaluated the anti-inflammatory potential of safety concentrations of NEWE using a copper sulfate (CuSO_4_) induced inflammation model in 3 days post-fertilization (dpf) larvae, this model utilizes the chemotactic properties of CuSO_4_ to disrupt neutrophil homeostasis, enabling real-time tracking of GFP-labeled neutrophil migration toward lateral line neuromasts - a validated method for quantifying inflammatory responses in zebrafish (Renshaw et al., 2006). As show in Figure 1A, neutrophils primarily localized to the caudal hematopoietic tissue in the control group, consistent with normal distribution patterns (Zhang et al., 2023b), but CuSO_4_ treatment prompted neutrophil migration towards the horizontal midline, where they formed clusters near the lateral line neuromasts (Figure 1B), indicating a pronounced inflammatory response. However, treatment with varying concentrations of NEWE effectively inhibited CuSO_4_-induced neutrophil migration in a does dependent manner, suggesting a potential anti-inflammatory activity (Figure 1B). Further, we investigated the effect of NEWE on expression of inflammatory genes using qRT-PCR, and found CuSO_4_ treatment significantly upregulated the expression of inflammatory genes, *interleukin 1 beta* (*il1b*), *nterleukin 11a* (*il11a*), *C-X-C motif chemokine ligand 8a* (*cxcl8a*), and *nuclear factor kappa B inhibitor alpha a* (*nfkbiaa*). Specifically, *il1b* expression increased by 37.22-fold, *il11a* by 9.67-fold, *cxcl8a* by 4.59-fold, and *nfkbiaa* by 3.91-fold compared to controls, respectively. However, treatment with NEWE could restore the CuSO_4_-indecued inflammatory gene expression. For example, in the presence of 100 μg/mL NEWE CuSO_4_ induced *il1b* expression was reduced to 8.78-fold from 37.22-fold (Figures 1C–F). Moreover, NEWE could restore the CuSO_4_-induced expression of *il11a*, *cxcl8a* and *nfkbiaa* to control level (Figures 1C–F). Collectively, our findings demonstrate that NEWE could effectively mitigate CuSO_4_-induced inflammation by reducing neutrophil recruitment to inflammatory site and downregulating inflammatory gene expression, highlighting its potential anti-inflammatory agent.

### 3.2 Antioxidant activity of NEWE

**FIGURE 2:**
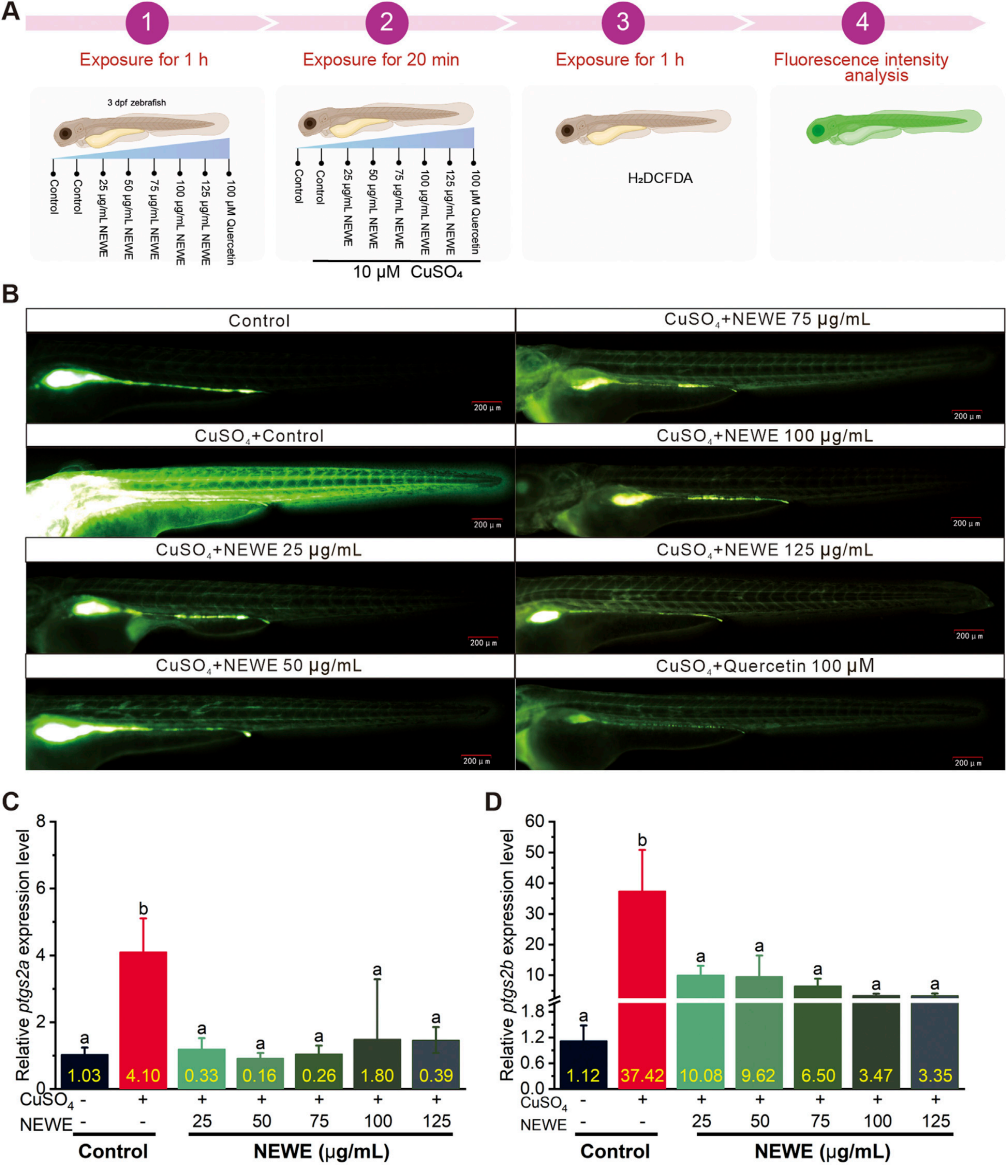
Fluorescence microscopy and qRT-PCR analysis of the antioxidant activity of *Nymphaea* “Eldorado” flower water extract (NEWE) in zebrafish larvae treated with CuSO_4_ in the presence and absence of NEWE or quercetin (positive control). **(A)** Flowchart of the experiments. **(B)** Fluorescence analysis of the antioxidant activity of NEWE in zebrafish larvae. The control (untreated) group shows baseline fluorescence. **(C, D)**. qRT-PCR analysis of the relative expression of oxidative stress-related genes *ptgs2a* and *ptgs2b*. Error bars represent standard deviations, and different letters indicate significant differences (*p* < 0.05).

The antioxidant activity of NEWE was assessed by fluorescence microscopy and qRT-PCR analysis. The DCFH-DA fluorescence probe operates through a redox-sensitive mechanism: non-fluorescent DCFH-DA passively diffuses across cell membranes and is deacetylated by intracellular esterases to form DCFH, which becomes fluorescent DCF upon oxidation by reactive oxygen species (ROS) (Zhang et al., 2023b). This oxidation process generates fluorescence intensity proportional to ROS levels, allowing semi-quantitative assessment of oxidative stress *in vivo* (Nguyen et al., 2020). Fluorescence imaging showed that CuSO_4_ remarkably increased oxidative stress in zebrafish larvae, whereas 100 μM quercetin (positive control) and different concentrations of NEWE reduced fluorescence intensity (Figures 2A,B), suggesting NEWE possesses antioxidant properties by reducing CuSO_4_-induced oxidative stress levels. The qRT-PCR results showed that CuSO_4_ exposure led to an upregulation of the oxidative stress-related genes, *ptgs2a* and *ptgs2b*, which encode prostaglandin synthase *2a* and *2b*, respectively (Ishikawa et al., 2007). Specifically, CuSO_4_ induced expression of *ptgs2a* to 4.1-fold, and *ptgs2b* to 37.42, relative to control, respectively. In contrast, NEWE treatment at 25–125 μg/mL effectively reversed these effects, reducing *ptgs2a* and *ptgs2b* expression (*p* < 0.05, Figures 2C,D). These findings demonstrate that NEWE mitigates CuSO_4_-induced oxidative stress in zebrafish larvae.

### 3.3 Transcriptomic analysis of NEWE’s anti-inflammatory mechanisms

**FIGURE 3:**
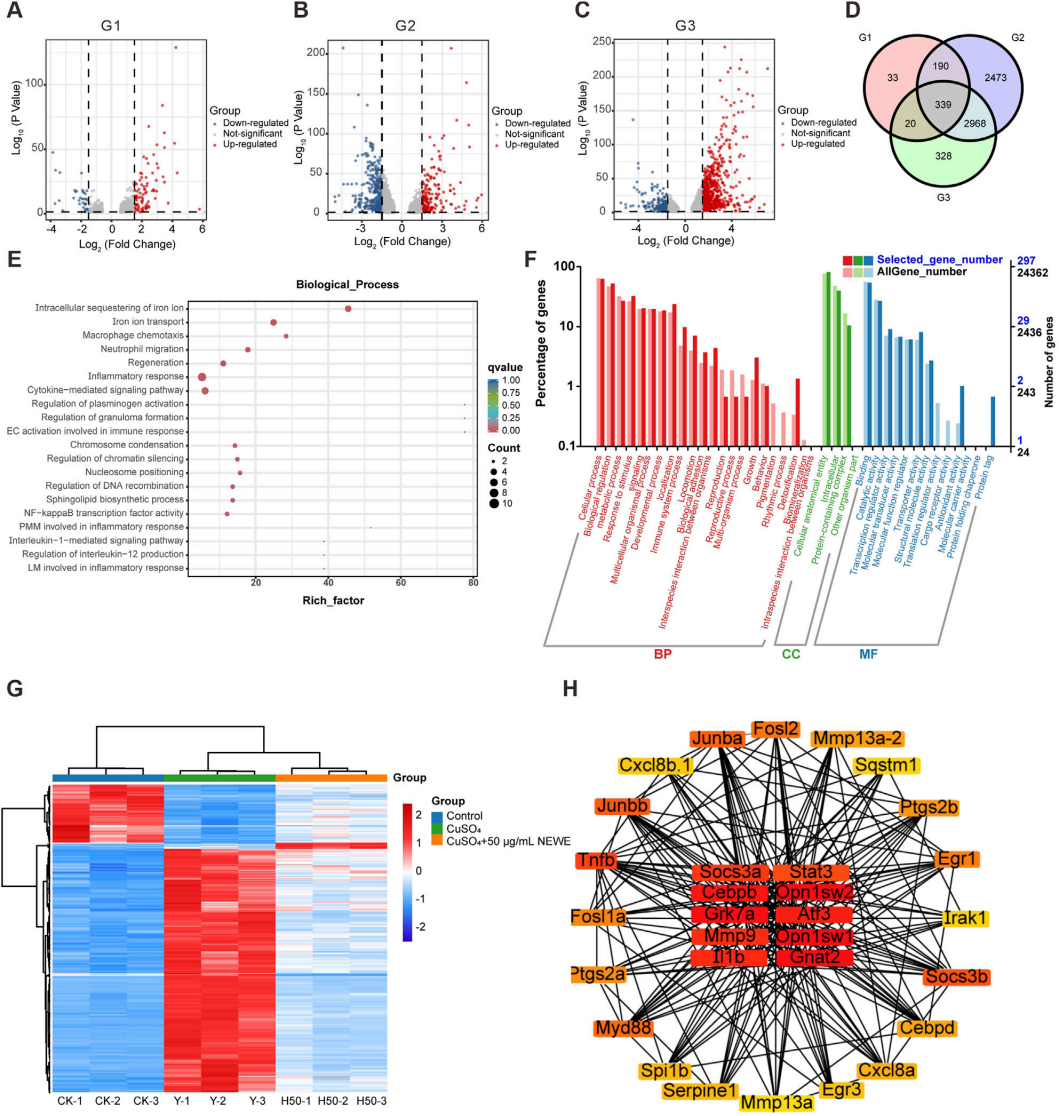
Differentially expressed genes (DEGs) and functional enrichment analyses of DEGs in zebrafish larvae treated with CuSO_4_ and *Nymphaea* “Eldorado” flower water extract. **(A-C)** DEGs in the control (untreated) group, model group (treated with CuSO_4_) vs control, and treatment group (treated with CuSO_4_ + 50 μg/mL NEWE) vs model group. Upregulated and downregulated genes are shown in red and blue, respectively. A total of 582, 5,970, and 3655 DEGs were identified in these groups. **(D)** DEGs shared between the groups. A total of 339 DEGs were shared across the three groups and were selected for further analysis. **(E)** Biological processes enriched in the selected DEGs. The size of each circle indicates the number of DEGs, and the color intensity represents the q-value. **(F)** Gene Ontology enrichment analysis of 339 DEGs. BP, biological process; CC, cellular component; MF, molecular function. **(G)** Expression profile of DEGs in each group. The color gradient from blue to red represents increasing expression levels. **(H)** Protein-protein interaction network of 30 hub DEGs, highlighting core interaction hubs with high connectivity. Nodes with low and high centrality are shown in yellow and red, respectively.

In order to examine the molecular mechanism of NEWE’s anti-inflammatory, we performed RNA-seq to profile global gene expression changes. Volcano plots showed that distinct gene expression patterns across the control, model group (treated with CuSO_4_), and treatment groups (CuSO_4_ + 50 μg/mL NEWE) (Figures 3A–C). A total of 339 differentially expressed genes (DEGs) was shared across the three groups and were selected for further functional annotation (Figure 3D), genes previously validated by qRT-PCR - including *il1b* (*interleukin 1 beta*), *ptgs2a/b* (*prostaglandin-endoperoxide synthase 2*), and *cxcl8a* (*C-X-C motif chemokine ligand 8a*) - emerged as core regulatory nodes (Figure 3H). Gene Ontology (GO) analysis showed that the selected DEGs were enriched in several biological processes, cellular components, and molecular functions (Figures 3E,F). Notably, genes associated with inflammatory response were significantly enriched, indicating their potential role in inflammatory pathways. Additionally, genes related to antioxidant activity were differentially expressed (Figures 2C,D), suggesting that antioxidant mechanisms may help mitigate inflammation by NEWE. Thus, future research should screen for DEGs related to both inflammatory and antioxidant activities to elucidate molecular mechanisms underlying NEWE’s anti-inflammatory effects. A heatmap showed that the expression profiles of these DEGs varied between different treatments (Figure 3G). A protein-protein interaction (PPI) network analysis indetified 30 hub DEGs as potential targets of NEWE, of particular significance, *stat3*, *serpine1*, and *mmp9* formed a tightly interconnected subnetwork that bridges inflammatory signaling and extracellular matrix reorganization (Figure 3H). These hub genes exhibited strong co-expression patterns with qRT-PCR validated targets, confirming their central role in NEWE-mediated anti-inflammatory responses.

### 3.4 Chemical composition of NEWE and network pharmacology analysis

**FIGURE 4:**
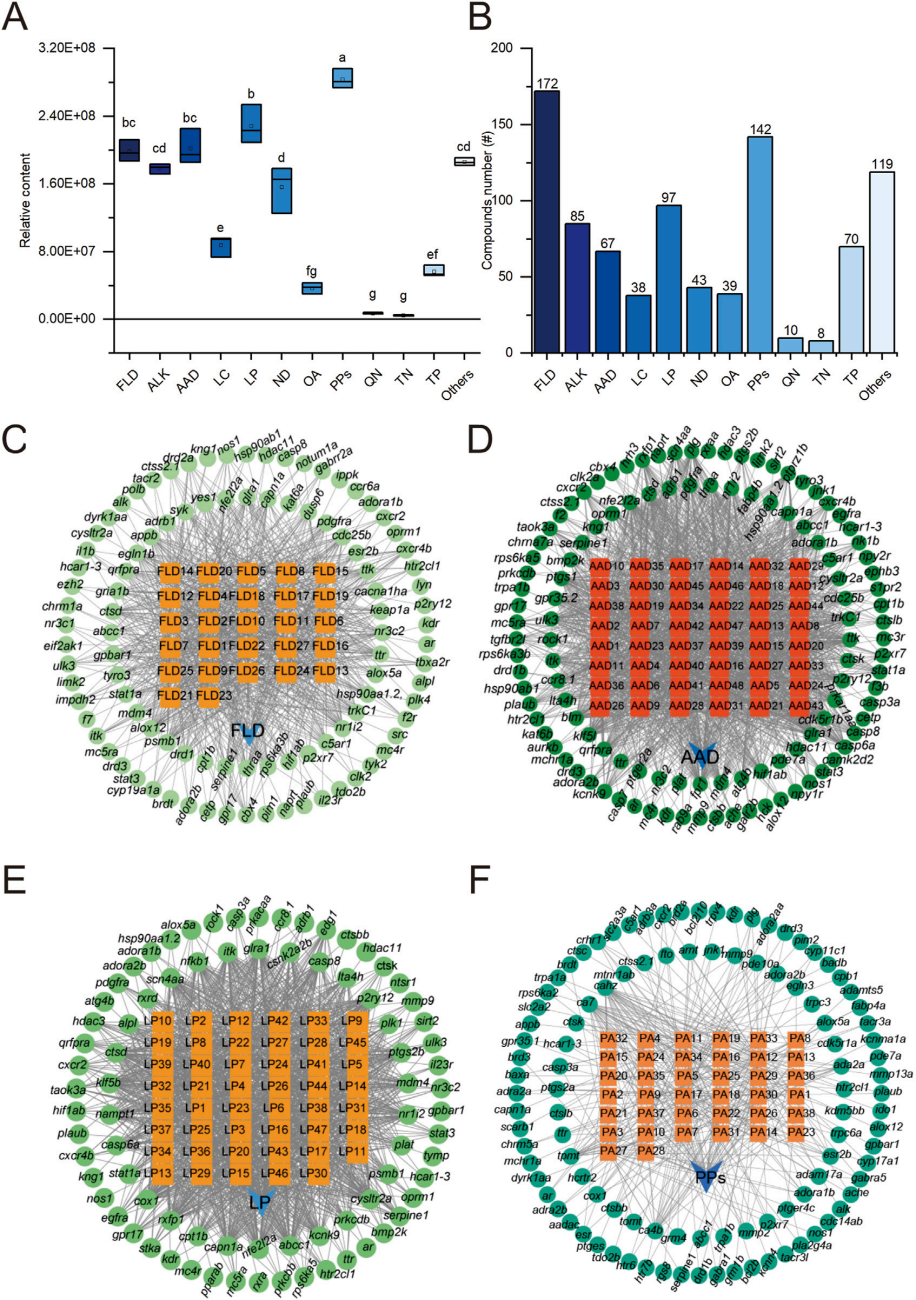
Network pharmacology analysis of major chemical constituents found in *Nymphaea* “Eldorado” flower water extract (NEWE) and their interactions with zebrafish target genes. **(A)** Relative abundance of major chemical constituents in NEWE. Different letters indicate significant differences (*p* < 0.05). **(B)** Number of unique chemical compounds in each category. **(C–F)** Interactions between chemical compounds (orange nodes) and target genes (green nodes). Abbreviations: FLD, flavonoids; ALK, alkaloids; AAD, amino acid derivatives; LC, lactones; LP, lipids; ND, nucleosides and nucleotides; OA, organic acids; PPs, phenylpropanoids; QN, quinones; TN, tannins; TP, terpenoids.

To further understand the molecular mechanism of NEWE in anti-inflammatory pathway, widely targeted metabolomic analysis was employed to identify potential compounds involved in NEWE-mediated anti-inflammatory (Supplementary Figure S2). A total of 891 compounds were identified and quantified, revealing a high degree of chemical diversity in the extract (Figures 4A,B). Flavonoids (172 compounds), alkaloids (85 compounds), lipids (97 compounds), and phenylpropanoids (142 compounds) were predominant. Subsequent network pharmacology analysis predicted potential biochemical interactions between these chemical compounds and target genes (Figures 4C–F; Supplementary Tables S2-S5). These interactions indicate the potential pharmacological relevance of these compounds and the importance of understanding these interactions to elucidate NEWE’s pharmacological effects.

### 3.5 Integrated multi-omics and network pharmacology reveal a polypharmacological hub gene network mediating NEWE’s anti-inflammatory activity

**FIGURE 5:**
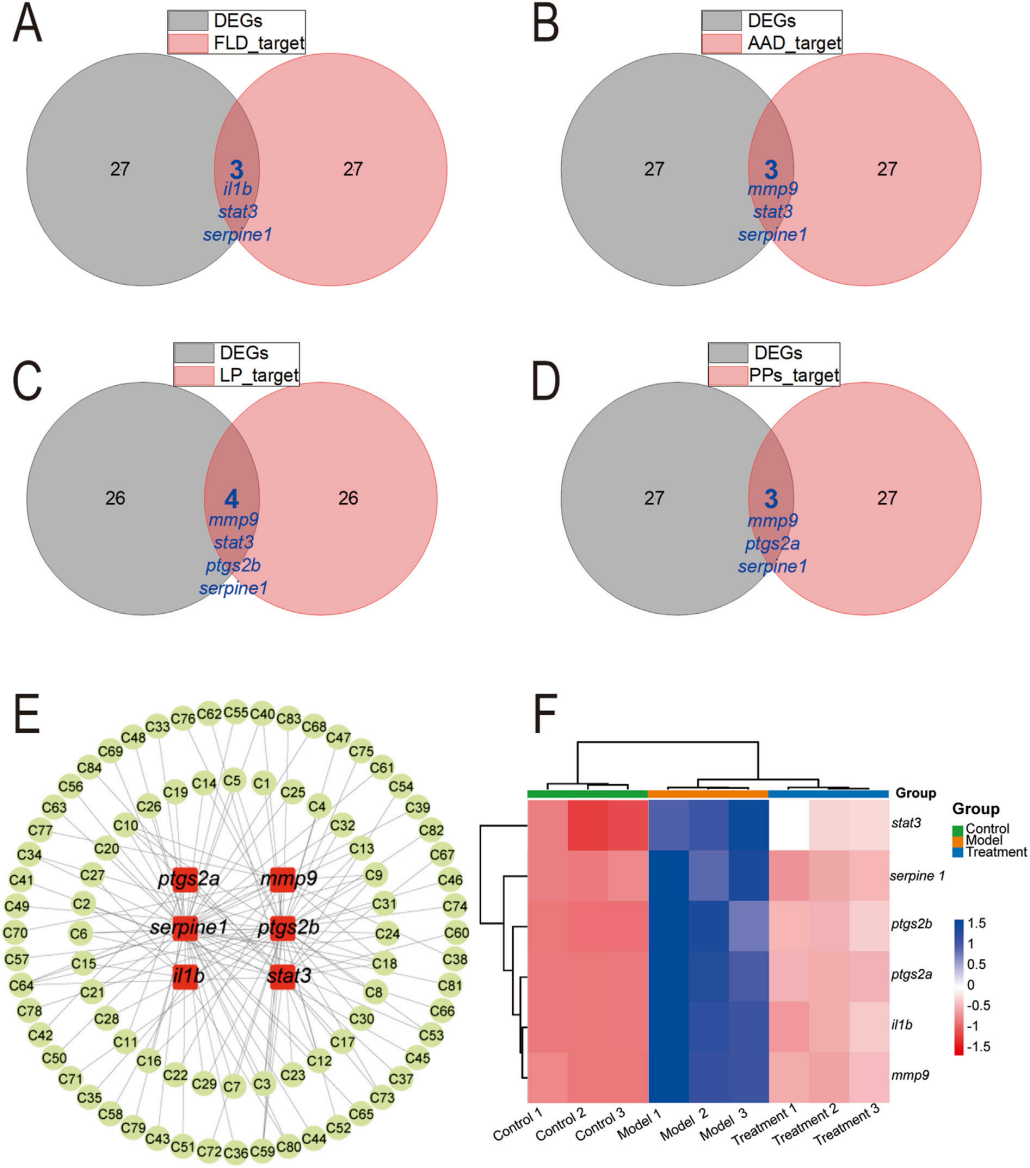
Analysis of hub differentially expressed genes (DEGs) associated with treatment with *Nymphaea* “Eldorado” flower water extract (NEWE) and genes targeted by four chemical compounds found in NEWE. **(A–D)** Overlap between DEGs and core target genes. **(E)** Interactions between six target genes (*il1b*, *stat3*, *serpine1*, *mmp9*, *ptgs2a*, and *ptgs2b*) (red nodes) and chemical compounds (green nodes). **(F)** Expression profile of target genes in different groups based on RNA-seq analysis. The color gradient from blue to red indicates increasing expression levels. Abbreviations: FLD, flavonoids; AAD, amino acid derivatives; LP, lipids; PPs, phenylpropanoids.

To investigate the potential mechanisms by which NEWE’s diverse chemical components exert their anti-inflammatory effects at the molecular level, we conducted an integrated analysis of our metabolomics and transcriptomics data, focusing on the identified hub DEGs and their predicted interactions with the various classes of compounds present in NEWE. Venn diagrams (Figures 5A–D) showed that among the 30 hub DEGs associated with NEWE treatment, several genes were consistently targeted by different classes of compounds within NEWE, including flavonoids, amino acid derivatives, lipids, and phenylpropanoids. Specifically, 6 key genes, including *il1b*, *stat3*, *serpine1*, *mmp9*, *ptgs2a*, and *ptgs2b* showed overlap, and the recurring presence across multiple compound-targeted groups suggests that these genes play pivotal roles in the response to NEWE treatment. Figure 5E depicts a network pharmacology map linking these six genes to various chemical constituents, underscoring their potential importance in mediating NEWE’s therapeutic effects. Additionally, RNA-seq expression profiling (Figure 5F) shows that the expression patterns of these core genes were distinct across the control, model, and treatment groups and that treatment normalized the gene expression disrupted by CuSO_4_ exposure. These findings collectively suggest that the six hub genes (*il1b*, *stat3*, *serpine1*, *mmp9*, *ptgs2a*, and *ptgs2b*) form a coordinated regulatory network through which multiple bioactive compounds in NEWE exert polypharmacological effects, simultaneously targeting inflammatory signaling, oxidative stress responses, and extracellular matrix remodeling. This multi-target engagement mechanism provides a molecular rationale for the observed anti-inflammatory efficacy of NEWE and highlights these genes as potential biomarkers for evaluating plant-derived therapeutics.

### 3.6 Expression of core target genes

To validate the transcriptional regulation of key inflammatory mediators identified through multi-omics integration, we quantified the expression dynamics of six core target genes using FPKM (Fragments Per Kilobase per Million mapped reads) values derived from RNA-seq data. This normalized metric accounts for both transcript length and sequencing depth, providing a reliable measure of relative gene expression levels across experimental groups. Upon treatment with CuSO_4_, six inflammatory genes were significantly upregulated: *ptgs2a* increased from 9.16 to 72.62 FPKM (approximately 7.9-fold), *serpine 1* from 6.81 to 43.64 FPKM (around 6.4-fold), *il1b* from 0.42 to 50.39 FPKM (about 120-fold), *mmp9* from 11.31 to 245.92 FPKM (approximately 21.7-fold), *ptgs2b* from 2.35 to 76.95 FPKM (around 32.8-fold), and *stat3* from 10.85 to 26.90 FPKM (approximately 2.5-fold). Notably, *il1b* exhibited the most dramatic induction, consistent with its role as a primary cytokine driver in copper-induced inflammation. Treatment with NEWE reversed these effects, significantly reducing gene expression in comparison to the CuSO_4_ group. These results indicate that NEWE possesses a potential anti-inflammatory effect, mitigating the overexpression induced by CuSO_4_.

### 3.7 Molecular docking of Serpine1-targeting compounds and functional validation of transcriptional regulation

To validate the network pharmacology prediction that Serpine1 (plasminogen activator inhibitor-1) acts as a critical mediator of NEWE’s bioactivity, we conducted molecular docking analyses on three representative compounds identified in the metabolomic profile: L-pyroglutamic acid, monomyristin, and protocatechuic acid (Supplementary Table S2). As illustrated in Figure 7, docking revealed specific interactions within the Serpine1 substrate-binding cleft: L-pyroglutamic acid formed hydrogen bonds with Thr-183, Arg-348, and Lys-235 (binding energy = −2.67 kcal/mol); monomyristin bound Asp-209 and Lys-311, positioning its acyl chain in the hydrophobic S4 pocket (binding energe = −0.67 kcal/mol); protocatechuic acid engaged Asp-209 and Asn-243 via hydrogen bonds (binding energy = −1.26 kcal/mol). These residues are critical for the structural integrity of the reactive center loop.

The functional relevance of these predicted interactions was assessed by measuring the compounds’ effects on *serpine1* gene expression in the zebrafish inflammation model. Treatment with L-pyroglutamic acid (0.20 mg/mL) significantly downregulated *serpine1* expression (Figure 7G). Similarly, protocatechuic acid induced a dose-dependent and significant downregulation at concentrations of 0.01, 0.06, and 0.10 mg/mL (Figure 7I). In contrast, monomyristin treatment did not significantly alter *serpine1* expression levels (Figure 7H).

**FIGURE 6:**
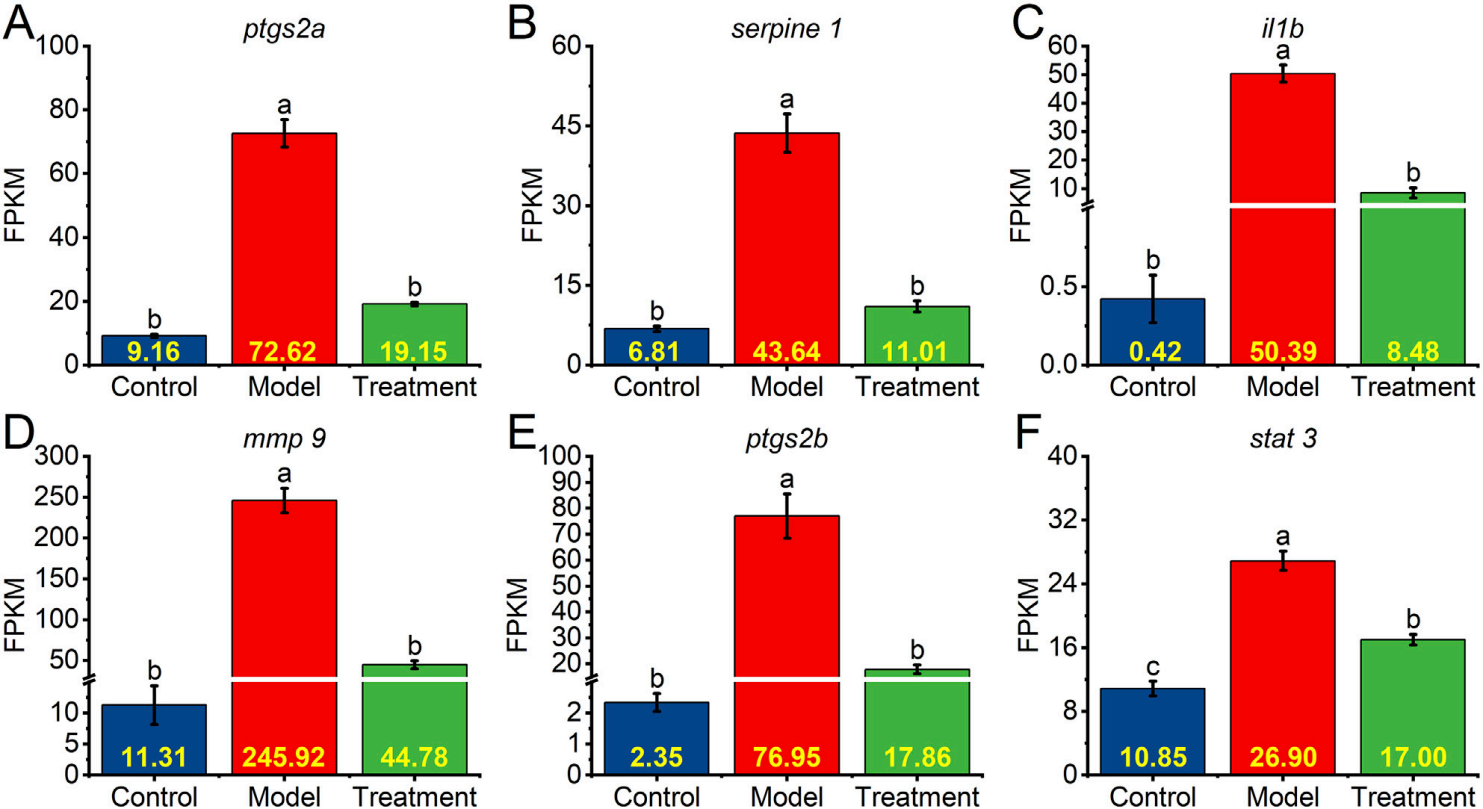
Expression levels of core target genes in different groups. Relative transcript levels were calculated as fragments per kilobase of exon per million fragments mapped (FPKM). **(A–F)** Relative expression of *ptgs2a*, *serpine1*, *il1b*, *mmp9*, *ptgs2b*, and *stat3* in the control (untreated) group, model group (treated with CuSO_4_), and treatment group (treated with CuSO_4_ and NEWE). Different letters indicate significant differences (*p* < 0.05). Data are means and standard errors of the mean.

The significant downregulation of *serpine 1* by L-pyroglutamic acid and protocatechuic acid provides functional biological evidence supporting their predicted interaction with Serpine1 through molecular docking, aligning with the 6.4-fold serpine1 downregulation observed with whole NEWE extract (Figure 6B). This suggests these compounds contribute to NEWE’s anti-inflammatory effect, potentially through direct or indirect interference with Serpine1 function or its regulation.

## 4 Discussion

This study demonstrated that *Nymphaea* “Eldorado” flower water extract (NEWE) exerts significant anti-inflammatory and antioxidant in a zebrafish model of copper sulfate-induced inflammation. The results provide a basis for using these natural bioactive compounds as inflammatory agents. Chronic inflammation is highly prevalent and impairs health-related quality of life. Thus, these compounds can help reduce the burden of inflammatory diseases (Hatch-McChesney and Smith, 2023; Pasiakos et al., 2016).

NEWE significantly reduced CuSO_4_-induced inflammation in zebrafish larvae, evidenced by a decrease in neutrophil migration and downregulation of the inflammatory genes *il1b*, *il11a*, *cxcl8a*, and *nfkbiaa*. Notably, these genes exhibited characteristic expression patterns in response to CuSO_4_ exposure, with *il1b* showing a remarkable 37.22-fold, consistent with their established roles as canonical inflammatory markers in zebrafish models (Barbalho et al., 2016; Bucher et al., 2018). These findings suggest that NEWE components attenuate acute inflammatory responses and thus can be used as anti-inflammatory agents.

Oxidative stress promotes inflammation and tissue damage, contributing to the progression of inflammatory diseases (Agnihotri et al., 2008; Alam et al., 2021; Bondia-Pons et al., 2012; Vo et al., 2011). Oxidative/ROS production assay and qRT-PCR analyses showed that NEWE treatment significantly reduced oxidative stress and inflammation in CuSO_4_-treated zebrafish larvae. The dual modulation of *ptgs2a/b* genes, which encode cyclooxygenase-2 isoforms critical for prostaglandin synthesis, further establishes their utility as oxidative stress biomarkers in this model. Specifically, NEWE attenuated neutrophil recruitment to inflammatory sites and downregulated key inflammatory genes, suggesting the inhibition of the oxidative stress-inflammation cascade triggered by CuSO_4_ exposure. These findings highlight the therapeutic potential of NEWE, as its antioxidative and anti-inflammatory effects may protect against inflammatory damage.

Transcriptomic analysis showed that NEWE treatment downregulated pro-inflammatory pathways and genes, including *il1b*, *stat3*, *serpine1*, *mmp9*, *ptgs2a*, and *ptgs2b*. *mmp9* was mainly expressed in neutrophil (Duan et al., 2024), the identification of *mmp9* as a differentially expressed gene (21.7-fold induction by CuSO_4_) is particularly noteworthy. Oxidative stress and inflammatory factors can upregulate the expression of *serpine1* (PAI-1) (Simone et al., 2014), the 6.4-fold upregulation of *serpine1* suggesting these genes may serve as secondary markers for chronic inflammatory progression. These results were corroborated by PPI network analyses and demonstrate NEWE’s potential to modulate inflammatory pathways, including those related to cytokine signaling and immune response regulation.

Widely targeted metabolomic analysis identified a diverse range of bioactive compounds, including flavonoids, alkaloids, amino acid derivatives, and phenylpropanoids. Flavonoids and phenylpropanoids have anti-inflammatory and antioxidant properties (Al-Khayri et al., 2022; Hasnat et al., 2024; Mykhailenko et al., 2021; Wang et al., 2022). Network pharmacology analysis showed that these compounds interacted with inflammatory genes, providing a molecular basis for the anti-inflammatory effects. The multi-target engagement observed - particularly the concurrent modulation of *il1b*, *stat3*, and *ptgs2a/b* - suggests these genes form a core biomarker network for evaluating anti-inflammatory efficacy. This polypharmacological approach mirrors recent strategies in complex disease biomarker discovery (Antolin et al., 2016; McDermott et al., 2013; Sun et al., 2019), where combined gene expression profiles outperform single-marker analyses. Understanding these interactions is crucial since multi-target therapeutics can have synergistic activity and reduce the side effects of single-target therapies.

The transcriptomic analysis revealed that NEWE treatment significantly attenuated CuSO_4_-induced inflammatory responses through coordinated downregulation of key pro-inflammatory mediators, including *ptgs2a*, *serpine1*, *il1b*, *mmp9*, *ptgs2b*, and *stat3*. This gene cluster demonstrates high diagnostic specificity for metal-induced inflammation, as evidenced by their minimal baseline expression (e.g., *il1b* FPKM 0.42 in controls vs. 50.39 in CuSO_4_ group) and strong response attenuation by NEWE. The hierarchical regulation observed - from early cytokine response genes (*il1b*) to downstream effectors (*mmp9*, *serpine1*) - provides a temporal framework for monitoring inflammatory progression. This comprehensive suppression of inflammatory pathway components underscores the extract’s potential as a therapeutic agent for inflammation-related pathologies.

**FIGURE 7:**
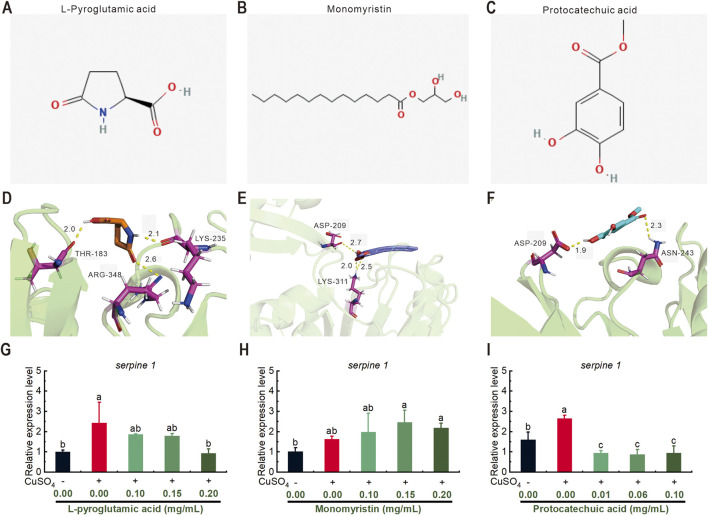
Molecular structure of three chemical compounds found in *Nymphaea* “Eldorado” flower water extract (NEWE) and their interactions with Serpine1 protein. **(A–C)** Molecular structure of L-pyroglutamic acid, monomyristin, and protocatechuic acid. **(D–F)** Interaction of these compounds with Serpine1. **(D)** L-pyroglutamic acid forms hydrogen bonds with Thr-183, Arg-348, and Lys-235. **(E)** Monomyristin establishes hydrogen bonds with Asp-209 and Lys-311. **(F)** Protocatechuic acid forms hydrogen bonds with Asp-209 and Asn-243. Molecular distances are shown in angstroms. Interacting residues in Serpine1 are shown in purple. **(G–I)** qRT-PCR analysis showing the relative expression of *serpine1* following treatment with the three compounds. Error bars represent standard deviations, and different letters indicate significant differences (*p* < 0.05).

Molecular docking revealed that key NEWE constituents -L-pyroglutamic acid, protocatechuic acid, monomyristin-exhibit binding affinities towards the active site of Serpine1, suggesting potential direct modulation of its function. This finding aligns with established bioactivities of these compounds: L-pyroglutamic acid demonstrates anti-inflammatory properties by suppressing LPS-induced NO production and exhibits neuroprotective effects in NGF-induced PC-12 cell differentiation (Gang et al., 2018). Protocatechuic acid possesses multi-faceted biological activities, including antioxidant, anti-inflammatory, and anti-hyperglycemic effects, mediated through interactions with diverse molecular targets (Lin et al., 2009). Monomyristin is characterized by significant antimicrobial and antifungal activities (Jumina et al., 2018). To functionally validate the predicted interactions with Serpine1 within our inflammatory context, we assessed the effects of individual compounds on serpine1 expression in our zebrafish inflammation model. Notably, L-pyroglutamic acid (0.20 mg/mL) significantly downregulated serpine1 transcription (Figure 7G), while protocatechuic acid induced dose-dependent suppression at 0.01, 0.06, and 0.10 mg/mL (Figure 7I). In contrast, monomyristin showed no significant effect (Figure 7H), highlighting compound-specific regulatory roles despite shared docking potential. These results align with transcriptomic data showing 6.4-fold serpine1 downregulation by NEWE and reinforce its role as a key pharmacological target.

The central position of Serpine1(PAI-1) within our multi-omics network is mechanistically well supported. Serpine1 functions as a critical node linking inflammation, fibrosis, and oxidative stress (Liu, 2008). Its overexpression drives pathological processes such as collagen accumulation following inflammatory lung injury (Eitzman et al., 1996). Conversely, Serpine1 modulates key pathways: it controls abdominal aortic aneurysm formation via TGF-β/Smad2/3 signaling (Zhao et al., 2025), influences macrophage polarization to reduce cardiac fibrosis in inflammatory cardiomyopathy, and suppresses inflammation in experimental autoimmune encephalomyelitis (EAE) by inhibiting Th1 cell secretion of IFNγ and TNFα (Akbar et al., 2023). Our data extend this established paradigm to copper-induced inflammation, suggesting an evolutionarily conserved pro-inflammatory role for Serpine1.

However, while transcriptional and docking data strongly implicate Serpine1, functional validation of its necessity remains essential. Future studies should employ CRISPR/Cas9-mediated serpine1 knockout in zebrafish or siRNA silencing in mammalian macrophages (e.g., RAW264.7) to definitively establish whether Serpine1 inhibition is sufficient to recapitulate NEWE’s anti-inflammatory effects. Complementary protein-level assays (e.g., ELISA for Serpine 1, MMP9, IL-1β) are equally critical to confirm translational relevance of observed mRNA changes.

## 5 Conclusion

This comprehensive study establishes *Nymphaea* “Eldorado” flower water extract (NEWE) as a multifaceted therapeutic agent targeting inflammation-oxidative stress crosstalk through integrated molecular mechanisms. Key findings reveal that NEWE (25–100 μg/mL) anti-inflammatory effects by suppressing CuSO_4_-induced neutrophil migration and downregulating critical proinflammatory genes, notably reducing *il1b* expression by 8.78-fold and *ptgs2a/b* expression by 25.63- and 11.17-fold, respectively.

Transcriptomic profiling identified 339 differentially expressed genes, with hierarchical suppression of *il1b* (120-fold reduction), *mmp9* (21.7-fold), and *serpine1* (6.4-fold) forming a core anti-inflammatory signature. Network pharmacology revealed polypharmacological interactions between 891 identified metabolites and inflammation-related targets, particularly flavonoids and phenylpropanoids targeting the *il1b*-*stat3*-*mmp9* axis. Molecular docking validated high-affinity binding of protocatechuic acid and L-pyroglutamic acid to Serpine1’s active site, providing structural evidence for pathway-specific modulation.

These findings systematically demonstrate that NEWE operates through a Serpine1-centric mechanism, coordinating transcriptional regulation of cytokine signaling (*stat3*, *il1b*), extracellular matrix remodeling (*mmp9*), and prostaglandin biosynthesis (*ptgs2a/b*). The multi-omics convergence of transcriptomic, metabolomic, and pharmacodynamic data positions NEWE as a promising candidate for developing plant-derived therapeutics against chronic inflammatory pathologies, offering advantages over conventional NSAIDs through its multi-target efficacy and reduced toxicity profile. Future studies should prioritize clinical validation of these zebrafish-derived mechanisms in mammalian systems and isolation of specific bioactive fractions for therapeutic optimization.

## Data Availability

The data presented in the study are deposited in the NCBI repository, accession number PRJNA1282926.
