# Supplementary material for: Nymphaea “Eldorado” flower extract targets serpine 1 to attenuate inflammatory and antioxidant crosstalk in zebrafish
Source: Front Pharmacol. 2025 Jul 11;16:1612233. doi: 10.3389/fphar.2025.1612233 (PMC12290299; doi:10.3389/fphar.2025.1612233)
Supplement: Supplementary file 1 [file DataSheet2.pdf]

A

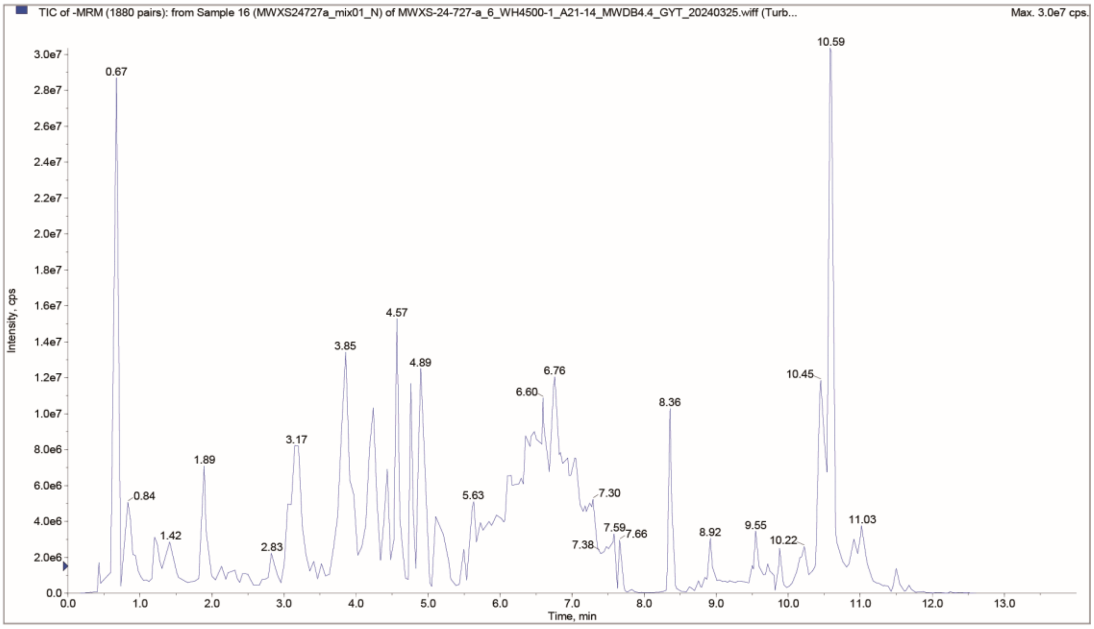

B

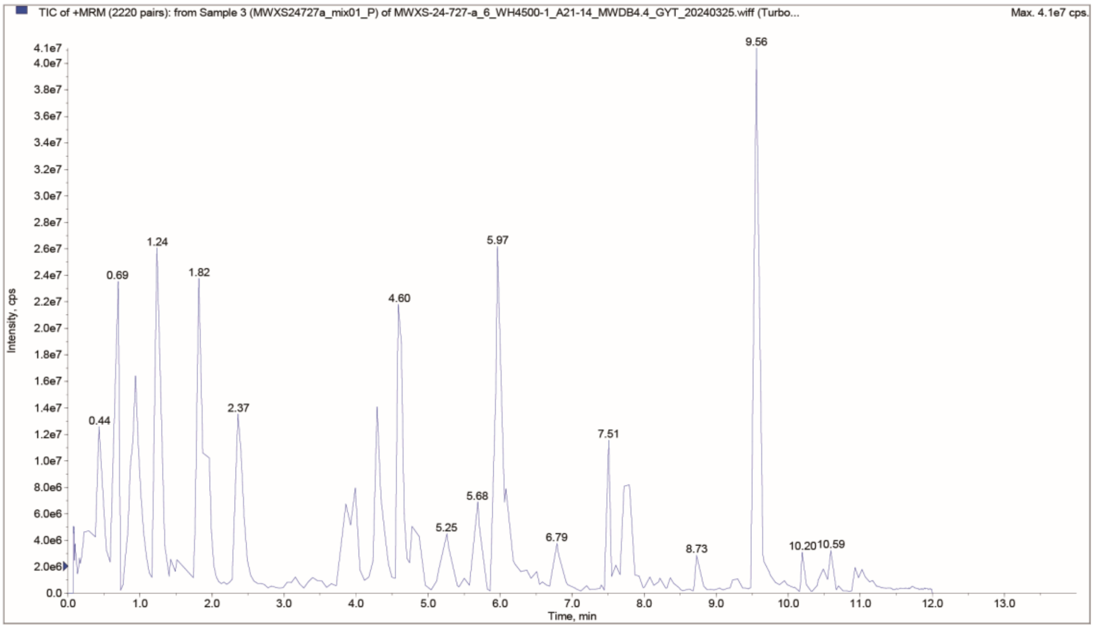

Figure S2. Total Ion Chromatogram (TIC) of NEWE mixed sample analyzed by mass spectrometry. (A) Negative ion mode of NEWE at a concentration of 50 μg/mL. (B) Positive ion mode of NEWE at a concentration of 50 μg/mL.
