# Supplementary material for: Nymphaea “Eldorado” flower extract targets serpine 1 to attenuate inflammatory and antioxidant crosstalk in zebrafish
Source: Front Pharmacol. 2025 Jul 11;16:1612233. doi: 10.3389/fphar.2025.1612233 (PMC12290299; doi:10.3389/fphar.2025.1612233)
Supplement: Supplementary file 4 [file DataSheet1.pdf]

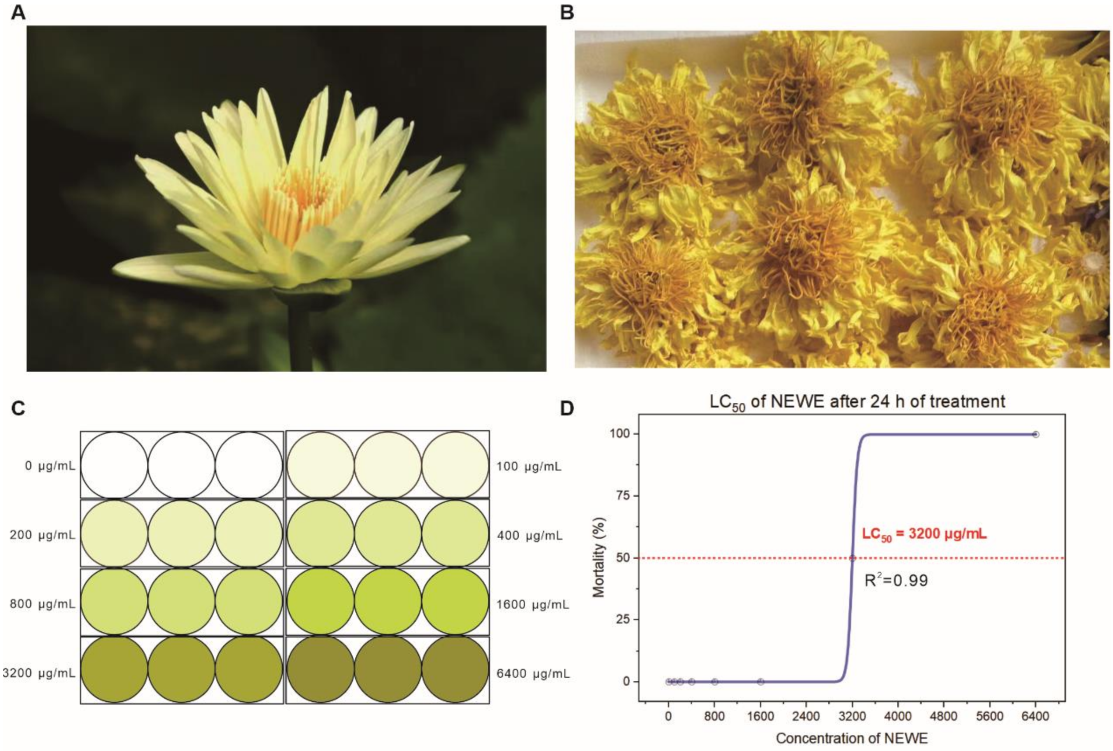


**Figure 1 S1. Flower characteristics of Nymphaea 'Eldorado' and the NEWE toxicity assay.**
(A) Fresh flower of *Nymphaea* 'Eldorado'. (B) Dried flowers of *Nymphaea* 'Eldorado'. (C) Schematic diagram of the zebrafish safety assay at 3 days post-fertilization (3dpf) used to assess the effects of NEWE. (D) Dose-lethality curve showing the relationship between NEWE concentration and lethality in 3dpf zebrafish after 24 hours of exposure, with an LC50 value of 3200 μg/mL and $R^2$ = 0.99.
